# Exploring the influence of endoscopist characteristics and artificial intelligence on adenoma detection in colonoscopy

**DOI:** 10.3389/fmed.2025.1720617

**Published:** 2026-01-12

**Authors:** Chenxia Zhang, Dexin Gong, Xiao Tao, Li Huang, Zehua Dong, Jie Pan, Jiejun Lin, Huang Su, Lianlian Wu

**Affiliations:** 1 Department of Gastroenterology, Renmin Hospital of Wuhan University, Wuhan, China; 2 Hubei Provincial Clinical Research Center for Digestive Disease Minimally Invasive Incision, Renmin Hospital of Wuhan University, Wuhan, China; 3 Key Laboratory of Hubei Province for Digestive System Disease, Renmin Hospital of Wuhan University, Wuhan, China; 4 Department of Gastroenterology, The First Affiliated Hospital, Zhejiang University School of Medicine, Hangzhou, China; 5 Department of Gastroenterology, Wenzhou Central Hospital, Wenzhou, Zhejiang, China; 6 Early Cancer Institute, University of Cambridge, Cambridge, United Kingdom

**Keywords:** adenoma detection rate, artificial intelligence, colonoscopy, endoscopist sex, polyp detection rate

## Abstract

**Background:**

Adenoma detection rate (ADR) is a key indicator of colonoscopy quality, but operator variability persists. Female clinicians may perform differently in other medical contexts, and artificial intelligence (AI) has been proposed to enhance detection. This study evaluated the impact of endoscopist characteristics and AI assistance on ADR in a real-world cohort.

**Methods:**

This retrospective cohort study analyzed colonoscopy data from 17,604 patients in a single center in China between January 2021 and August 2022. Endoscopist characteristics were determined via survey, and AI-assisted procedures were identified based on implementation dates. The primary outcome was ADR; secondary outcomes included polyp detection rate (PDR), lesion size and location, and detection rates of advanced adenomas (AA) and sessile serrated lesions (SSL). Multivariable regression models were employed to adjust for confounders, including patient and endoscopist characteristics.

**Results:**

Of the 17,604 colonoscopies, 12,638 (71.8%) were performed by male endoscopists and 4,966 (28.2%) by female endoscopists. After adjustment by nine confounders at patient and endoscopist levels and the use of AI, female endoscopists achieved higher ADR (AOR 1.273, 95% CI 1.171 to 1.385; *p* < 0.001) and PDR (AOR 1.816, 95% CI 1.679 to 1.964; *p* < 0.001). Female endoscopists also outperformed in detecting diminutive adenomas and lesions in the proximal colon. AI assistance improved ADR among male endoscopists (AOR 1.166, 95% CI 1.064 to 1.278; *p* = 0.001) but had no significant effect for females. With AI, ADR among male endoscopists increased to 28.77%, approaching the level observed in female endoscopists without AI (30.05%).

**Conclusion:**

Endoscopist characteristics, particularly sex and experience, and AI assistance influence ADR. Female endoscopists demonstrated superior performance in detecting lesions during colonoscopy, especially for small and proximally located lesions. AI assistance significantly enhanced the performance of male endoscopists, reducing sex-based disparities in ADR.

## Introduction

The quality of colonoscopy is essential for effective colorectal cancer (CRC) screening and prevention (1). The adenoma detection rate (ADR) is a key indicator of colonoscopy quality, as lower ADR has been consistently associated with a higher risk of post-colonoscopy CRC and related mortality (2, 3). Despite its clinical importance, substantial variation in ADR persists among endoscopists, and the determinants of this variability are not fully understood (4). While operator expertise and procedural experience are recognized contributors, evidence regarding other endoscopist characteristics remains limited and inconclusive (5–7).

Sociodemographic factors such as clinician sex have been shown to influence clinical practice patterns and patient outcomes (8). For example, studies in surgical populations suggest that female clinicians may demonstrate distinct practice patterns, such as greater adherence to guidelines and more patient-centered approaches, which are associated with improved outcomes (9–11). Whether similar sex-related differences exist in endoscopic performance remains uncertain, and available data on their impact on ADR are scarce.

In addition to demographic and professional factors, artificial intelligence (AI) has recently been introduced to enhance the quality of colonoscopy. Randomized controlled trials have demonstrated that AI-assisted colonoscopy improves ADR, particularly among endoscopists with lower baseline performance (12–15). As an objective tool, AI has the potential to reduce operator-related variability by providing real-time visual support for polyp detection (16). However, whether the effectiveness of AI is consistent across different endoscopist subgroups, or whether its impact is modified by operator characteristics such as sex or experience, remains uncertain.

Against this background, the present study aimed to identify factors associated with ADR in a real-world cohort. Both patient-level and endoscopist-level characteristics, as well as the use of AI assistance, were analyzed. Specifically, this study sought to: first, identify the most influential determinants of ADR in routine practice; and second, explore whether the effect of AI assistance on ADR varies across endoscopist subgroups.

## Materials and methods

### Study design and data sources

This retrospective observational cohort study was conducted in the Department of Gastroenterology at Wenzhou Central Hospital, a tertiary care center in Zhejiang Province, China. Clinical data were collected from patients who underwent colonoscopy between January 1, 2021, and August 10, 2022, to investigate the association between endoscopist characteristics and lesion detection. Between June 1 and October 31, 2021, computer-aided detection (CADe) devices were gradually implemented at the hospital. During this transitional phase, endoscopists underwent standardized training to perform colonoscopy with AI assistance. From November 1, 2021, all endoscopic systems were fully equipped with CADe and the system was routinely activated at the beginning of each workday. Colonoscopy procedures are assigned according to the daily scheduling roster of the endoscopy unit. Endoscopists are allocated based on predefined duty schedules and physician availability. This study utilized the same dataset as a previously published study that explored the effect of AI on adenoma detection (17).

To ensure a clear comparison between AI-assisted and non-AI-assisted procedures, the transitional phase (June 1 to October 31, 2021), during which AI was variably used, was excluded from the analysis. Patients were also excluded if they were younger than 18 years old; underwent colonoscopy for known or suspected malignancy; had a history of colorectal surgery, polyposis, intestinal tuberculosis, or inflammatory bowel disease; or if the colonoscopy failed to reach the cecum or was associated with inadequate bowel preparation. For patients who underwent multiple colonoscopies during the study period, only the first procedure was included in the analysis. The study was approved by the institutional review boards of Renmin Hospital of Wuhan University and Wenzhou Central Hospital. Written informed consent was waived due to the retrospective nature of the study. The study followed the Strengthening the Reporting of Observational Studies in Epidemiology (STROBE) reporting guideline.

### Exposure and outcomes

Endoscopist characteristics were determined through a *post hoc* questionnaire survey. The questionnaire collected information on endoscopists’ age, sex, years of endoscopic experience (≤5 years, 5–10 years, ≥10 years), and endoscopy volume (≤5,000; 5,000–10,000; ≥10,000). The primary outcome was the ADR. Secondary outcomes included the polyp detection rate (PDR); ADR stratified by adenoma size (diminutive [≤5 mm], small [5–10 mm], and large [≥10 mm]) and location (caecum, ascending colon, transverse colon, descending colon, sigmoid colon, and rectum); PDR stratified by polyp size and location; detection rates of advanced adenomas (AA) and sessile serrated lesions (SSL); as well as the number of adenomas per colonoscopy (APC) and polyps per colonoscopy (PPC). All detection rates were calculated as the proportion of patients with at least one endoscopically identified or histopathological confirmed lesion relative to the total number of colonoscopies performed. AA were defined as adenomas ≥10 mm in size (as documented by the endoscopist), those with high-grade dysplasia, adenomas with villous histology, or invasive carcinoma.

### Regression models

Univariate and multivariable logistic regression models were employed to evaluate the association between endoscopist characteristics and detection rates, adjusting for nine potential confounders at both the patient level (age, sex, colonoscopy indication, inpatient or outpatient status, and anesthesia use) and the endoscopist level (age, sex, years of endoscopic experience, endoscopy volume), as well as the use of AI assistance. Poisson regression models were used to analyze differences in APC and PPC.

### Statistical analysis

Descriptive statistics were used to summarize the baseline characteristics of the study cohort. Student’s *t*-test or the Mann–Whitney U test was used to compare continuous variables, while the chi-square (χ^2^) test or Fisher’s exact test was used for categorical variables, as appropriate. All statistical tests were two-sided, and a *p*-value < 0.05 was considered statistically significant. All analyses were conducted using SPSS 25 (IBM, Chicago, Illinois, United States).

## Results

### Baseline characteristics of the study cohort

As shown in Supplementary Figure S1, a total of 25,939 patients underwent colonoscopy during the study period. After excluding ineligible individuals, 17,604 eligible patients were included in the final analysis. Of these, 12,638 procedures (71.79%) were performed by male endoscopists and 4,966 (28.21%) by female endoscopists. Table 1 presents the baseline characteristics of the study cohort and logistic regression analyses of factors associated with ADR. In multivariable analysis, older patient age (AOR 1.055, 95% CI 1.052 to 1.058; *p* < 0.001), inpatient recruitment (AOR 0.887, 95% CI 0.813 to 0.969; *p* = 0.008), sedation use (AOR 1.308, 95% CI 1.125 to 1.521; *p* < 0.001), female endoscopist sex (AOR 1.273, 95% CI 1.171 to 1.385; *p* < 0.001), ≥10 years of endoscopic experience (AOR 1.387, 95% CI 1.153 to 1.669; *p* < 0.001), and AI assistance (AOR 1.121, 95% CI 1.039 to 1.210; *p* = 0.003) were significantly associated with higher ADR.

**Table 1:** Baseline characteristics and logistic regression analysis of factors associated with ADR.

Variables	Total (*n* = 17,604)	Univariate analysis		Multivariate analysis	
		*p*-value	OR (95%CI)	*p*-value	AOR (95%CI)
Patient age, Mean ± SD	52.52 ± 12.57	<0.001	1.051 (1.048–1.054)	<0.001	1.055 (1.052–1.058)
Patient gender, *n* (%)					
Male	8,728 (49.58)		1.000 (Reference)		1.000 (Reference)
Female	8,876 (50.42)	<0.001	0.538 (0.504–0.575)	<0.001	0.493 (0.460–0.529)
Indication for colonoscopy, *n* (%)					
Screening	9,304 (52.85)		1.000 (Reference)		1.000 (Reference)
Diagnostic	7,773 (44.15)	0.211	0.958 (0.897–1.024)	0.037	0.926 (0.861–0.995)
Surveillance	527 (2.99)	0.067	1.191 (0.988–1.436)	0.179	0.872 (0.714–1.065)
Recruitment, *n* (%)					
Outpatient	14,049 (79.81)		1.000 (Reference)		1.000 (Reference)
Inpatient	3,555 (20.19)	<0.001	1.256 (1.161–1.360)	0.008	0.887 (0.813–0.969)
Sedation use, *n* (%)					
No	1,064 (6.04)		1.000 (Reference)		1.000 (Reference)
Yes	16,540 (93.96)	0.352	0.938 (0.819–1.074)	<0.001	1.308 (1.125–1.521)
Physician age, Mean ± SD	42.96 ± 5.74	0.013	1.007 (1.001–1.013)	0.037	0.992 (0.985–0.999)
Endoscopist gender, *n* (%)					
Male	12,638 (71.79)		1.000 (Reference)		1.000 (Reference)
Female	4,966 (28.21)	<0.001	1.155 (1.075–1.241)	<0.001	1.273 (1.171–1.385)
Years of endoscopic experience, *n* (%)					
≤5 years	1,779 (10.11)		1.000 (Reference)		1.000 (Reference)
5–10 years	3,736 (21.22)	0.174	0.914 (0.803–1.041)	0.369	0.937 (0.814–1.080)
≥10 years	12,089 (68.67)	<0.001	1.265 (1.130–1.416)	<0.001	1.387 (1.153–1.669)
Endoscopy volume, *n* (%)					
≤5,000	3,764 (21.38)		1.000 (Reference)		1.000 (Reference)
5,000–10,000	4,699 (26.69)	<0.001	1.290 (1.171–1.421)	0.168	1.105 (0.959–1.273)
≥10,000	9,141 (51.93)	<0.001	1.281 (1.175–1.397)	0.874	1.014 (0.855–1.203)
AI assistance, *n* (%)					
No	5,390 (30.61)		1.000 (Reference)		1.000 (Reference)
Yes	12,214 (69.38)	0.003	1.113 (1.037–1.196)	0.003	1.121 (1.039–1.210)

SD, Standard deviation; OR, unadjusted odds ratio; AOR, adjusted odds ratio; CI, confidence interval.

### Association between endoscopist sex and detection rates

**Figure 1:**
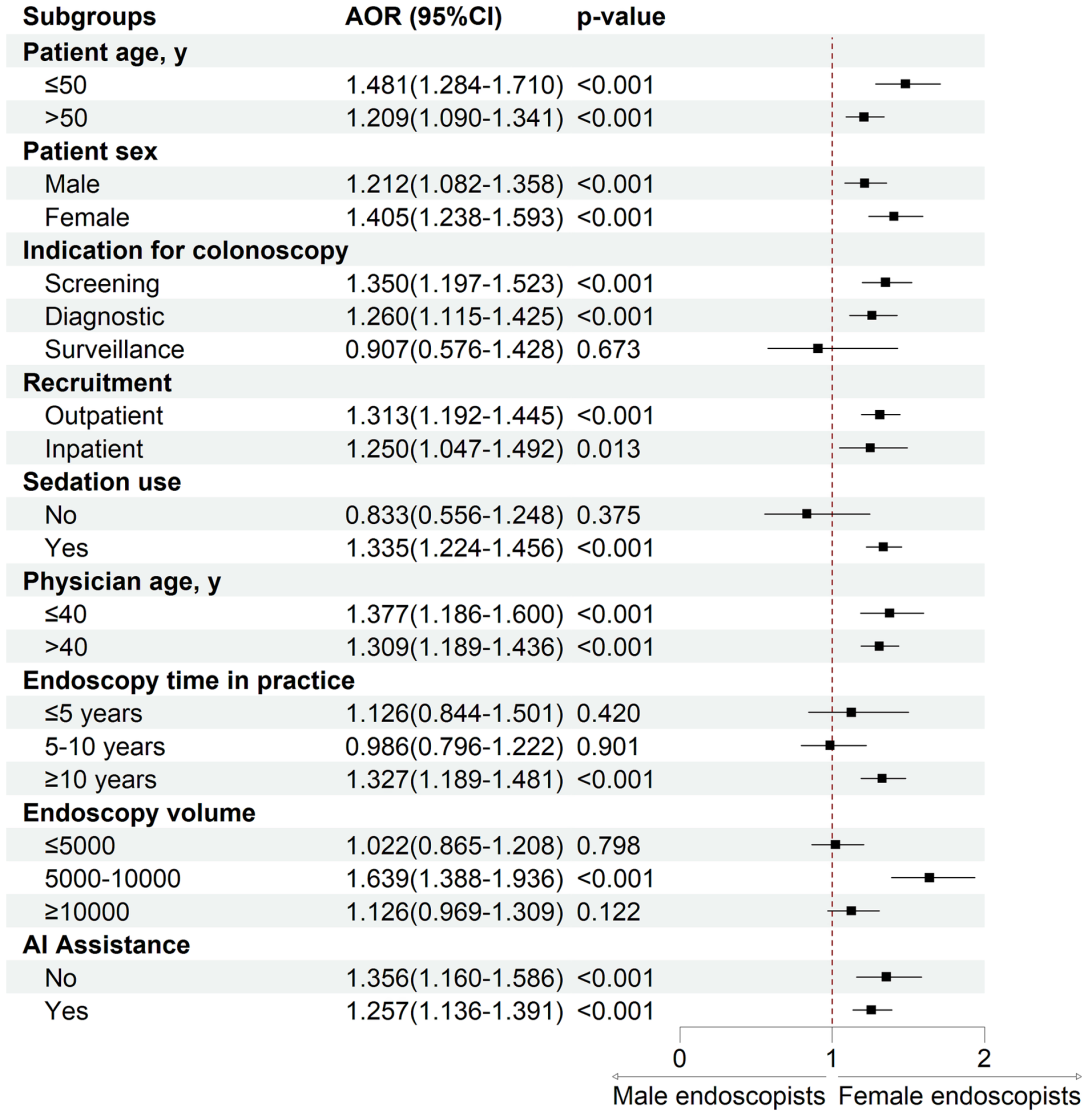
Subgroup analysis of the effect of endoscopist sex on adenoma detection rate.

As presented in Table 2, after adjusting for AI assistance and covariates related to patient and endoscopist characteristics, female endoscopists demonstrated significantly higher ADR (30.97% vs. 27.98%; AOR 1.273, 95% CI 1.171 to 1.385; *p* < 0.001) and PDR (55.24% vs. 44.94%; AOR 1.816, 95% CI 1.679 to 1.964; *p* < 0.001) compared to their male counterparts. Regarding lesion characteristics, female endoscopists detected more diminutive adenomas (23.74% vs. 20.33%; AOR 1.353, 95% CI 1.236 to 1.482; *p* < 0.001) and polyps smaller than 10 mm (53.69% vs. 42.89%; AOR 1.562, 95% CI 1.409 to 1.732; *p* < 0.001). In terms of lesion location, a higher proportion of adenomas were detected in the proximal colon by female endoscopists (19.47% vs. 16.31%; AOR 1.390, 95% CI 1.213 to 1.594; *p* < 0.001). There were no statistically significant differences in detection rates of AA (5.74% vs. 5.58%; *p* = 0.251) or SSL (0.60% vs. 0.53%; *p* = 0.323) between male and female endoscopists. Subgroup analyses revealed that female endoscopists consistently outperformed males in adenoma detection across various strata, including patient age, sex, colonoscopy indication, inpatient or outpatient status, anesthesia use, as well as endoscopist age, experience, procedure volume, and AI assistance (Figure 1). Subgroup analyses for other lesion detection indicators are presented in Supplementary Table S1.

**Table 2:** The effect of endoscopist sex on detection rate.

Variables	Endoscopist		Univariate analysis		Multivariate analysis	
	Male (*n* = 12,638)	Female (*n* = 4,966)	*p*-value	OR (95%CI)	*p*-value	AOR (95%CI)
ADR	3,536 (27.98)	1,538 (30.97)	<0.001	1.155 (1.075–1.241)	<0.001	1.273 (1.171–1.385)
PDR	5,679 (44.94)	2,743 (55.24)	<0.001	1.512 (1.415–1.615)	<0.001	1.816 (1.679–1.964)
Advanced adenoma detection rate	705 (5.58)	285 (5.74)	0.677	1.031 (0.894–1.187)	0.251	1.098 (0.936–1.288)
Sessile serrated lesion detection rate	67 (0.53)	30 (0.60)	0.551	1.140 (0.740–1.756)	0.323	1.277 (0.787–2.073)
Adenoma size						
Diminutive (≤5mm)	2,569 (20.33)	1,179 (23.74)	<0.001	1.220 (1.128–1.320)	<0.001	1.353 (1.236–1.482)
Small (>5 to <10mm)	1,163 (9.20)	527 (10.61)	0.004	1.171 (1.051–1.306)	<0.001	1.251 (1.107–1.414)
Large (≥10mm)	624 (4.94)	243 (4.89)	0.903	0.991 (0.851–1.153)	0.495	1.061 (0.895–1.258)
Adenoma location						
Cecum	184 (1.46)	103 (2.07)	0.004	1.434 (1.124–1.829)	<0.001	1.631 (1.232–2.161)
Ascending colon	893 (7.07)	452 (9.10)	<0.001	1.317 (1.170–1.482)	<0.001	1.467 (1.283–1.678)
Transverse colon	1,168 (9.24)	575 (11.58)	<0.001	1.286 (1.157–1.429)	<0.001	1.447 (1.282–1.633)
Descending colon	665 (5.26)	260 (5.24)	0.944	0.995 (0.859–1.153)	0.283	1.095 (0.928–1.293)
Sigmoid colon	1,251 (9.90)	544 (10.95)	0.037	1.120 (1.007–1.246)	0.006	1.184 (1.050–1.336)
Rectum	477 (3.77)	209 (4.21)	0.18	1.120 (0.949–1.322)	0.284	1.107 (0.919–1.335)
Polyp size						
Diminutive (≤5mm)	4,690 (37.11)	2,419 (48.71)	<0.001	1.610 (1.506–1.720)	<0.001	1.922 (1.777–2.078)
Small (>5 to <10mm)	1,628 (12.88)	741 (14.92)	<0.001	1.186 (1.080–1.303)	<0.001	1.277 (1.148–1.420)
Large (≥10mm)	757 (5.99)	308 (6.20)	0.595	1.038 (0.905–1.190)	0.154	1.118 (0.959–1.303)
Polyp location						
Cecum	457 (3.62)	240 (4.83)	<0.001	1.354 (1.154–1.588)	<0.001	1.556 (1.296–1.867)
Ascending colon	1,455 (11.51)	810 (16.31)	<0.001	1.498 (1.365–1.644)	<0.001	1.684 (1.514–1.873)
Transverse colon	1,843 (14.58)	1,023 (20.60)	<0.001	1.520 (1.396–1.654)	<0.001	1.728 (1.566–1.906)
Descending colon	1,057 (8.36)	539 (10.85)	<0.001	1.334 (1.196–1.488)	<0.001	1.549 (1.368–1.754)
Sigmoid colon	2,295 (18.16)	1,214 (24.45)	<0.001	1.458 (1.348–1.578)	<0.001	1.704 (1.538–1.888)
Rectum	1,530 (12.11)	900 (18.12)	<0.001	1.607 (1.469–1.758)	<0.001	1.325 (1.143–1.536)
APC, mean (SD)	0.39 (0.73)	0.46 (0.83)	<0.001	1.182 (1.113–1.255)	<0.001	1.302 (1.224–1.386)
PPC, mean (SD)	1.02 (1.91)	1.61 (2.81)	<0.001	1.582 (1.493–1.679)	<0.001	1.724 (1.623–1.831)

OR, Unadjusted odds ratio; AOR, Adjusted odds ratio; CI, Confidence interval.

### Impact of AI assistance on endoscopists’ detection rates

Table 3 summarizes the impact of AI assistance on lesion detection rates during colonoscopy. Of all procedures, 12,214 (69.40%) were performed with AI assistance. After adjusting for patient- and endoscopist-level covariates, AI assistance was associated with a significantly higher ADR (29.49% vs. 27.31%; AOR 1.121, 95% CI 1.038 to 1.210; *p* = 0.003) and PDR (48.41% vs. 46.55%; AOR 1.093, 95% CI 1.019 to 1.172; *p* = 0.013). However, AI assistance had no significant effect on detection rates of AA (5.58% vs. 5.71%, *p* = 0.358) or SSL (0.52% vs. 0.63%, *p* = 0.386). Compared to the non-AI group, AI primarily enhanced the detection of diminutive adenomas and diminutive polyps. Regarding lesion location, AI assistance increased detection rates of adenomas (10.48% vs. 8.59%; AOR 1.248, 95% CI 1.111 to 1.401; *p* < 0.001) and polyps (17.03% vs. 14.58%; AOR 1.210, 95% CI 1.1021 to 1.328; *p* < 0.001) in the transverse colon.

**Table 3:** The influence of AI on endoscopists’ detection rates.

Variables	AI Assistance		Univariate analysis		Multivariate analysis	
	No (*n* = 5,390)	Yes (*n* = 12,214)	*p*-value	OR (95%CI)	*p*-value	AOR (95%CI)
ADR	1,472 (27.31)	3,602 (29.49)	0.003	1.113 (1.037–1.196)	0.003	1.121 (1.038–1.210)
PDR	2,509 (46.55)	5,913 (48.41)	0.023	1.078 (1.011–1.149)	0.013	1.093 (1.019–1.172)
Advanced adenoma detection rate	308 (5.71)	682 (5.58)	0.729	0.976 (0.850–1.121)	0.358	0.935 (0.810–1.079)
Sessile serrated lesion detection rate	34 (0.63)	63 (0.52)	0.343	0.817 (0.538–1.241)	0.386	0.828 (0.541–1.267)
Adenoma size						
Diminutive (≤5mm)	1,083 (20.09)	2,665 (21.82)	0.010	1.110 (1.025–1.201)	0.006	1.125 (1.035–1.222)
Small (>5 to <10mm)	477 (8.85)	1,213 (9.93)	0.025	1.136 (1.016–1.269)	0.058	1.118 (0.996–1.255)
Large (≥10mm)	267 (4.95)	600 (4.91)	0.907	0.991 (0.855–1.149)	0.488	0.947 (0.813–1.104)
Adenoma location						
Cecum	81 (1.50)	206 (1.69)	0.375	1.124 (0.868–1.457)	0.238	1.172 (0.900–1.526)
Ascending colon	407 (7.55)	938 (7.68)	0.767	1.018 (0.902–1.149)	0.915	1.007 (0.888–1.141)
Transverse colon	463 (8.59)	1,280 (10.48)	<0.001	1.246 (1.114–1.393)	<0.001	1.248 (1.111–1.401)
Descending colon	278 (5.16)	647 (5.30)	0.702	1.029 (0.890–1.188)	0.779	1.021 (0.880–1.185)
Sigmoid colon	521 (9.67)	1,274 (10.43)	0.122	1.088 (0.978–1.212)	0.168	1.081 (0.968–1.207)
Rectum	207 (3.84)	479 (3.92)	0.797	1.022 (0.865–1.207)	0.944	1.006 (0.849–1.193)
Polyp size						
Diminutive (≤5mm)	2,117 (39.28)	4,992 (40.87)	0.047	1.069 (1.001–1.141)	0.019	1.087 (1.014–1.167)
Small (>5 to <10mm)	688 (12.76)	1,681 (13.76)	0.074	1.091 (0.992–1.200)	0.176	1.071 (0.970–1.183)
Large (≥10mm)	327 (6.07)	738 (6.04)	0.950	0.996 (0.871–1.139)	0.486	0.952 (0.828–1.094)
Polyp location						
Cecum	205 (3.80)	492 (4.03)	0.481	1.062 (0.899–1.254)	0.369	1.081 (0.912–1.281)
Ascending colon	659 (12.23)	1,606 (13.15)	0.092	1.087 (0.986–1.197)	0.113	1.085 (0.981–1.200)
Transverse colon	786 (14.58)	2,080 (17.03)	<0.001	1.202 (1.100–1.314)	<0.001	1.210 (1.102–1.328)
Descending colon	479 (8.89)	1,117 (9.15)	0.582	1.032 (0.922–1.155)	0.494	1.041 (0.927–1.169)
Sigmoid colon	1,064 (19.74)	2,445 (20.02)	0.671	1.018 (0.939–1.103)	0.623	1.021 (0.939–1.111)
Rectum	718 (13.32)	1,712 (14.02)	0.217	1.061 (0.966–1.165)	0.247	1.059 (0.961–1.167)
APC, mean (SD)	0.39 (0.75)	0.42 (0.77)	0.009	1.085 (1.021–1.154)	0.012	1.079 (1.017–1.144)
PPC, mean (SD)	1.16 (2.27)	1.20 (2.19)	0.306	1.033 (0.971–1.098)	0.409	1.025 (0.967–1.087)

OR, Unadjusted odds ratio; AOR, Adjusted odds ratio; CI, Confidence interval.

**Figure 2:**
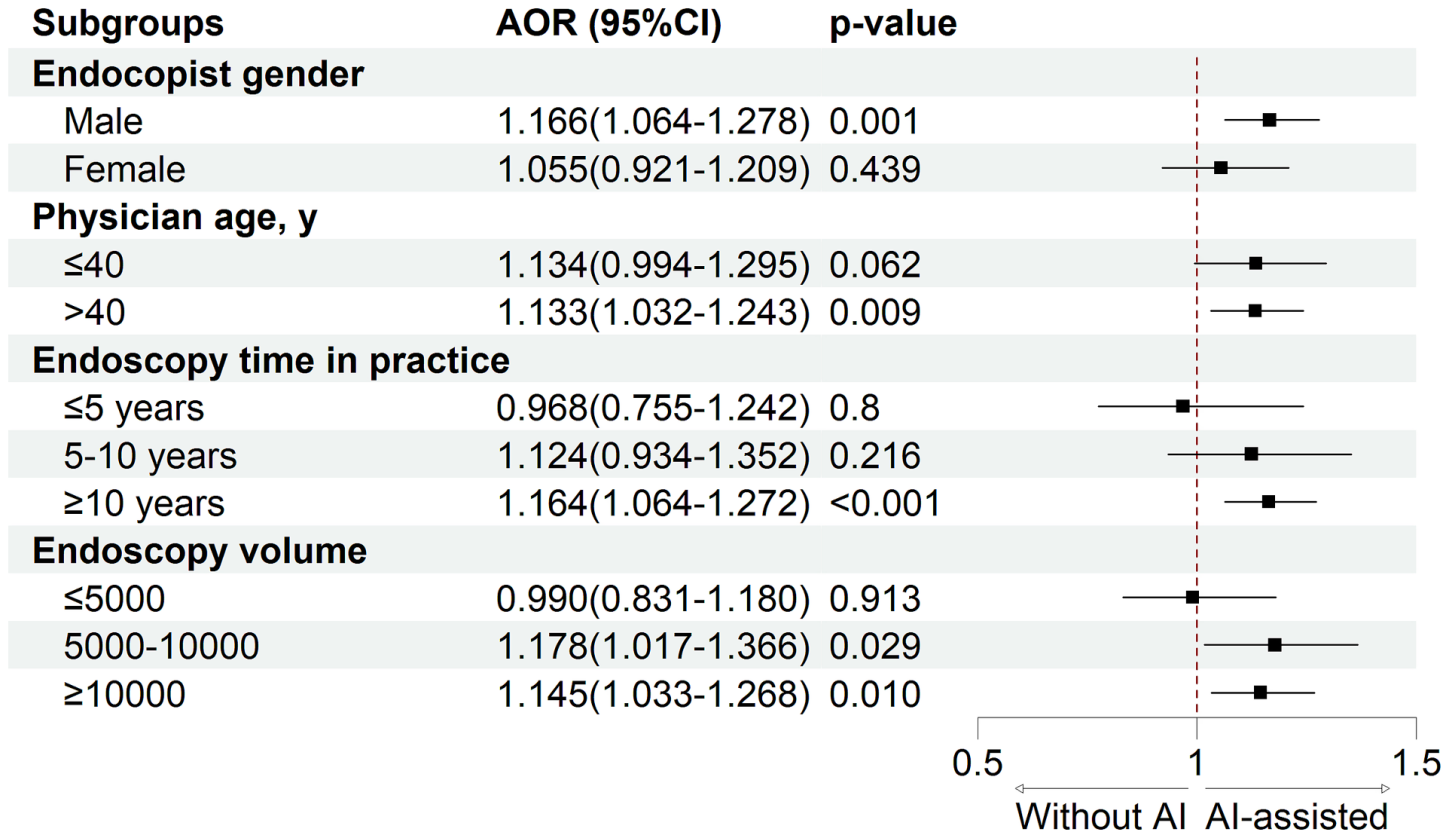
Subgroup analysis of the effect of AI on adenoma detection rate.

To further explore the impact of AI assistance on different endoscopist subgroups, we conducted stratified analyses based on endoscopist sex, age, endoscopic experience, and procedure volume (Figure 2). As shown in Supplementary Table S2, after adjusting for covariates at the patient and endoscopist levels, the results showed that AI significantly improved the ADR (28.77% vs. 26.06%; AOR 1.166, 95% CI 1.064 to 1.278; *p* = 0.001) and PDR (45.75% vs. 42.97%; AOR 1.136, 95% CI 1.046 to 1.234; *p* = 0.002) among male endoscopists. In contrast, no significant improvements were observed among female endoscopists (ADR: 31.45% vs. 30.05%, *p* = 0.439; PDR: 55.68% vs. 54.37%, *p* = 0.714). Additionally, AI assistance notably improved detection rates among endoscopists with extensive endoscopic experience, whereas no significant differences in ADR (24.36% vs. 25.65%; *p* = 0.780) or PDR (40.09% vs. 39.81%; *p* = 0.624) were observed among less experienced endoscopists.

### Interaction between endoscopist sex and AI assistance

The preceding analyses indicated that female endoscopists generally outperformed their male counterparts in lesion detection, while AI assistance significantly improved the detection rates among male endoscopists. To further investigate the interaction between these two factors, we compared the detection performance of AI-assisted male endoscopists with that of female endoscopists without AI assistance, as presented in Table 4. With AI assistance, ADR among male endoscopists increased to 28.77%, approaching the level observed in female endoscopists without AI (30.05%; AOR 1.180, 95% CI 1.036 to 1.344; *p* = 0.012). However, PDR remained higher among female endoscopists (54.37% vs. 45.75%; AOR 1.508, 95% CI 1.299 to 1.752; *p* < 0.001). Further analysis revealed that this superior performance was particularly evident in the detection of diminutive lesions (ADR: 22.96% vs. 20.97%, *p* < 0.001; PDR: 47.70% vs. 37.81%, *p* < 0.001) and those located in the proximal colon.

**Table 4:** Comparison of detection rates between AI-assisted male endoscopists and female endoscopists without AI.

Variables	Male and AI (*n* = 8,942)	Female (*n* = 1,694)	Univariate analysis		Multivariate analysis	
			*p*-value	OR (95%CI)	*p*-value	AOR (95%CI)
ADR	2,573 (28.77)	509 (30.05)	0.290	1.063 (0.949–1.191)	0.012	1.180 (1.036 ~ 1.344)
PDR	4,091 (45.75)	921 (54.37)	<0.001	1.413 (1.273–1.568)	<0.001	1.711 (1.517 ~ 1.929)
Advanced adenoma detection rate	497 (5.56)	100 (5.90)	0.572	1.066 (0.854–1.330)	0.239	1.159 (0.907 ~ 1.481)
Sessile serrated lesion detection rate	44 (0.49)	11 (0.65)	0.409	1.322 (0.681–2.564)	0.640	1.192 (0.571 ~ 2.490)
Adenoma size						
Diminutive (≤5mm)	1,875 (20.97)	389 (22.96)	0.066	1.123 (0.992–1.272)	<0.001	1.281 (1.113 ~ 1.474)
Small (>5 to <10mm)	859 (9.61)	173 (10.21)	0.440	1.070 (0.901–1.271)	0.149	1.151 (0.951 ~ 1.392)
Large (≥10mm)	441 (4.93)	84 (4.96)	0.963	1.006 (0.792–1.278)	0.515	1.092 (0.838 ~ 1.423)
Adenoma location						
Cecum	136 (1.52)	33 (1.95)	0.199	1.286 (0.876–1.889)	0.021	1.641 (1.077 ~ 2.501)
Ascending colon	635 (7.10)	149 (8.80)	0.015	1.262 (1.047–1.520)	<0.001	1.427 (1.159 ~ 1.756)
Transverse colon	875 (9.79)	170 (10.04)	0.751	1.028 (0.865–1.223)	0.160	1.148 (0.947 ~ 1.391)
Descending colon	475 (5.31)	88 (5.19)	0.843	0.977 (0.773–1.234)	0.718	1.048 (0.811 ~ 1.355)
Sigmoid colon	908 (10.15)	178 (10.51)	0.660	1.039 (0.877–1.231)	0.174	1.138 (0.945 ~ 1.372)
Rectum	353 (3.95)	83 (4.90)	0.071	1.254 (0.981–1.602)	0.134	1.231 (0.938 ~ 1.614)
Polyp size						
Diminutive (≤5mm)	3,381 (37.81)	808 (47.70)	0.066	1.123 (0.992–1.272)	<0.001	1.836 (1.629 ~ 2.069)
Small (>5 to <10mm)	1,187 (13.27)	247 (14.58)	0.440	1.070 (0.901–1.271)	0.018	1.220 (1.035 ~ 1.438)
Large (≥10mm)	532 (5.95)	102 (6.02)	0.963	1.006 (0.792–1.278)	0.456	1.096 (0.861 ~ 1.395)
Polyp location						
Cecum	326 (3.65)	74 (4.37)	0.152	1.207 (0.933–1.562)	0.011	1.446 (1.089 ~ 1.920)
Ascending colon	1,052 (11.76)	256 (15.11)	<0.001	1.335 (1.152–1.548)	<0.001	1.522 (1.291 ~ 1.794)
Transverse colon	1,370 (15.32)	313 (18.48)	0.001	1.253 (1.094–1.434)	<0.001	1.439 (1.236 ~ 1.676)
Descending colon	752 (8.41)	174 (10.27)	0.013	1.247 (1.048–1.483)	<0.001	1.439 (1.187 ~ 1.744)
Sigmoid colon	1,636 (18.3)	405 (23.91)	<0.001	1.403 (1.240–1.588)	<0.001	1.580 (1.376 ~ 1.815)
Rectum	1,103 (12.34)	291 (17.18)	<0.001	1.474 (1.280–1.697)	<0.001	1.605 (1.372 ~ 1.878)
APC, mean (SD)	0.40 (0.74)	0.44 (0.81)	0.046	1.102 (1.002–1.212)	<0.001	1.196 (1.086 ~ 1.317)
PPC, mean (SD)	1.03 (1.90)	1.53 (2.83)	<0.001	1.489 (1.353–1.638)	<0.001	1.630 (1.482 ~ 1.792)

OR, unadjusted odds ratio; AOR, adjusted odds ratio; CI, confidence interval.

## Discussion

This large retrospective cohort study investigated factors associated with ADR during colonoscopy, focusing on endoscopist characteristics and AI assistance. We found that female endoscopists demonstrated significantly higher ADR and PDR than male endoscopists, particularly for diminutive adenomas and lesions located in the proximal colon. No significant sex-related differences were observed for larger adenomas. Additionally, AI assistance improved the ADR of male endoscopists, narrowing the performance gap associated with endoscopist sex.

Sex-related variations in clinical performance have been documented across multiple medical specialties, yet evidence in endoscopic practice remains limited (18–20). In the present study, female endoscopists maintained higher ADRs and PDRs even after adjustment for confounders, suggesting an independent association between operator sex and detection performance. This advantage was most evident for diminutive and proximal lesions, which are easily overlooked and demand greater visual precision (21, 22). Several procedural and cognitive mechanisms may underlie these differences. Previous studies have shown that endoscopists with more systematic withdrawal patterns, controlled scope manipulation, and stable mucosal visualization achieve higher ADRs (23, 24). Female endoscopists may adopt slower, more deliberate inspection strategies, particularly in anatomically complex regions such as the proximal colon, enhancing the likelihood of identifying subtle or flat lesions. Additionally, potential differences in attentional persistence and visual scanning behavior may influence the ability to recognize low-contrast mucosal abnormalities. Although these mechanisms remain speculative, the observed pattern underscores that procedural behaviors and perceptual strategies may drive the sex-related disparity in ADR. These findings highlight the need for performance-based training emphasizing meticulous mucosal inspection techniques to standardize colonoscopy quality across operators.

The integration of AI into colonoscopy has been widely recognized as an effective strategy to improve detection consistency and reduce human variability (25, 26). AI-assisted systems provide real-time visual prompts that augment human perception, thereby reducing missed lesions. Randomized controlled trials have demonstrated that AI-assisted colonoscopy improves ADR and reduces adenoma miss rates (13, 15). However, outcomes from real-world studies have been more heterogeneous, suggesting that AI performance may depend on operator characteristics, procedural environment, and baseline skill level (27–29). For instance, Wei et al. conducted a multicenter randomized controlled trial and found no statistically significant difference in ADR between the AI-assisted group and the control group (35.9% vs. 37.2%, *p* = 0.774), suggesting that AI systems may encounter challenges and limitations in practical application (29). In our study, we found that the performance of AI-assisted systems is influenced by the clinical context, which may help explain their more modest performance of AI in real-world scenarios compared to RCTs. These findings underscore the importance of optimizing implementation strategies to maximize the clinical utility of AI in endoscopic practice.

Our findings revealed that AI assistance provided significant benefits for male endoscopists but minimal additional improvement for female endoscopists, whose baseline ADRs were already high. This pattern indicates that AI may partially compensate for operator-related factors such as vigilance, scanning thoroughness, or real-time lesion recognition. However, when baseline procedural quality is already optimized, the incremental advantage of AI diminishes. Given that current AI systems primarily function by generating visual alerts to flag suspicious lesions, they may be particularly effective in compensating for inattentiveness or reduced vigilance during procedures, conditions potentially more prevalent among certain operators. Additionally, our findings showed that AI appeared to enhance ADR more effectively among senior endoscopists than among those with limited experience, aligning with prior evidence that experienced operators are better equipped to interpret and integrate AI feedback into procedural workflow (30). The limited benefit of AI among less experienced endoscopists may reflect the interaction between foundational technical skills and cognitive processing demands during colonoscopy. Novice operators must simultaneously manage scope navigation, mucosal exposure, and real-time decision-making, which may substantially increase cognitive load. Under such conditions, AI-generated visual alerts may compete for attentional resources rather than effectively augment lesion detection. In addition, over-reliance on AI prompts in the absence of systematic visual scanning strategies may further attenuate its additive value. In contrast, experienced endoscopists possess more automated technical skills, including stable scope control and effective fold-flattening, enabling seamless integration of AI assistance into established visual search patterns (31, 32). These observations reinforce the notion that AI should be viewed as an augmentative adjunct that enhances human capability rather than a substitute for fundamental technical skills.

Our results carry important clinical implications. The superior ADR achieved by female endoscopists highlights the potential role of cognitive and procedural behaviors in determining colonoscopy quality. Behavioral traits such as deliberate withdrawal, stable scope control, and sustained attentional focus may represent key performance attributes that could be emphasized in training and quality improvement initiatives. Furthermore, the selective benefit of AI across operator subgroups suggests that its implementation should be individualized. Tailoring AI deployment according to operator experience, baseline performance, and case complexity may optimize its clinical utility. Future AI systems incorporating adaptive learning algorithms and personalized feedback mechanisms could further minimize inter-operator disparities and enhance the overall quality of colonoscopy.

Several limitations of this study should be acknowledged. First, this study was conducted in a single tertiary center, and the relatively homogeneous patient population and practice environment may limit generalizability. Multicenter studies encompassing diverse clinical settings are warranted to confirm these findings. Second, despite multivariable adjustment, unmeasured confounders cannot be excluded given the retrospective design. Withdrawal time, a well-established determinant of adenoma detection, was unavailable in the present study. Longer withdrawal duration was associated with higher ADR and may contribute to inter-operator variability. It is possible that female endoscopists, on average, devote more time to mucosal inspection during withdrawal, which may partially contribute to their higher detection rates. Rather than negating the observed association, this consideration suggests that sex-related differences in ADR may be mediated through procedural behaviors such as inspection thoroughness, withdrawal technique, and time allocation, which are integral components of colonoscopy quality. Although direct assessment of this mechanism was not possible, the association between endoscopist sex and ADR remained robust after adjustment for multiple patient- and operator-level covariates. Third, although patient assignment followed the routine scheduling roster of the endoscopy unit rather than patient choice, allocation was not strictly randomized. Therefore, subtle selection bias related to unmeasured factors, such as patient compliance or procedural complexity, cannot be completely excluded.

In conclusion, this large real-world study demonstrates that both endoscopist characteristics and AI assistance significantly influence colonoscopy quality. Female endoscopists consistently achieved higher ADR and PDR, particularly for diminutive and proximal lesions. AI assistance significantly enhanced ADR among male endoscopists, effectively narrowing the sex-related performance gap. These findings underscore that AI can serve as a valuable adjunct to reduce operator variability and promote standardized, high-quality colonoscopy, while continued emphasis on procedural training remains indispensable. Future multicenter prospective studies are warranted to validate these findings and optimize the clinical integration of AI in gastrointestinal endoscopy.

## Data Availability

The data analyzed in this study is subject to the following licenses/restrictions: The data that support the findings of this study after deidentification are available from the corresponding author upon reasonable request. Requests to access these datasets should be directed to wu_leanne@whu.edu.cn.
