# Supplementary material for: Exploring the influence of endoscopist characteristics and artificial intelligence on adenoma detection in colonoscopy
Source: Front Med (Lausanne). 2026 Jan 12;12:1720617. doi: 10.3389/fmed.2025.1720617 (PMC12833059; doi:10.3389/fmed.2025.1720617)
Supplement: Supplementary file 1 [file Table_1.docx]

**Table S1. Subgroup analysis of the effect of endoscopist sex on detection rates**

| Variables | ADR | | | | PDR | | | | AA | | | | APC | | | | PPC | | | |
| --- | --- | --- | --- | --- | --- | --- | --- | --- | --- | --- | --- | --- | --- | --- | --- | --- | --- | --- | --- | --- |
|  | Male | Female | *P* | AOR(95%CI) | Male | Female | *P* | AOR(95%CI) | Male | Female | *P* | AOR(95%CI) | Male | Female | *P* | AOR(95%CI) | Male | Female | *P* | AOR(95%CI) |
| Patient age, y |  |  |  |  |  |  |  |  |  |  |  |  |  |  |  |  |  |  |  |  |
| ≤50 | 876  (16.22) | 460  (20.17) | <.001 | 1.481  (1.284-1.710) | 1730  (32.03) | 981  (43.01) | <.001 | 1.887  (1.677-2.122) | 124  (2.30) | 62  (2.72) | 0.324 | 1.192  (0.841-1.688) | 0.19  (0.48) | 0.26  (0.61) | <.001 | 1.490  (1.309-1.696) | 0.60  (1.39) | 0.93  (1.79) | <.001 | 1.697  (1.507-1.910) |
| >50 | 2260  (36.76) | 1078  (40.15) | <.001 | 1.209  (1.090-1.341) | 3949  (54.57) | 1762  (65.62) | <.001 | 1.821  (1.638-2.025) | 581  (8.03) | 223  (8.31) | 0.467 | 1.069  (0.893-1.281) | 0.53  (0.84) | 0.63  (0.94) | <.001 | 1.241  (1.150-1.340) | 1.33  (2.16) | 2.19  (3.35) | <.001 | 1.718  (1.594-1.851) |
| Patient gender |  |  |  |  |  |  |  |  |  |  |  |  |  |  |  |  |  |  |  |  |
| Male | 2199  (34.70) | 870  (36.39) | <.001 | 1.212  (1.082-1.358) | 3422  (54.00) | 1489  (62.28) | <.001 | 1.740  (1.556-1.947) | 456  (7.20) | 173  (7.24) | 0.529 | 1.067  (0.872-1.307) | 0.50  (0.83) | 0.58  (0.95) | <.001 | 1.265  (1.162-1.377) | 1.38  (2.28) | 2.12  (3.37) | <.001 | 1.659  (1.531-1.798) |
| Female | 1337  (21.22) | 668  (25.94) | <.001 | 1.405  (1.238-1.593) | 2257  (35.82) | 1254  (48.7) | <.001 | 1.957  (1.753-2.186) | 249  (3.95) | 112  (4.35) | 0.280 | 1.156  (0.888-1.506) | 0.28  (0.60) | 0.35  (0.67) | <.001 | 1.354  (1.220-1.504) | 0.66  (1.35) | 1.14  (2.06) | <.001 | 1.828  (1.651-2.023) |
| Indication for colonoscopy |  |  |  |  |  |  |  |  |  |  |  |  |  |  |  |  |  |  |  |  |
| Screening | 1884  (27.96) | 823  (32.09) | <.001 | 1.350  (1.197-1.523) | 3000  (44.52) | 1467  (57.19) | <.001 | 2.077  (1.855-2.326) | 344  (5.10) | 137  (5.34) | 0.306 | 1.132  (0.893-1.435) | 0.39  (0.73) | 0.47  (0.83) | <.001 | 1.308  (1.196-1.431) | 0.97  (1.80) | 1.71  (2.91) | <.001 | 1.901  (1.751-2.064) |
| Diagnostic | 1528  (27.65) | 666  (29.64) | <.001 | 1.260  (1.115-1.425) | 2497  (45.19) | 1185  (52.74) | <.001 | 1.638  (1.462-1.836) | 320  (5.79) | 134  (5.96) | 0.359 | 1.113  (0.885-1.399) | 0.38  (0.72) | 0.44  (0.82) | <.001 | 1.296  (1.174-1.430) | 1.06  (2.01) | 1.47  (2.61) | <.001 | 1.525  (1.381-1.685) |
| Surveillance | 124  (33.24) | 49  (31.82) | 0.673 | 0.907  (0.576-1.428) | 182  (48.79) | 91  (59.09) | 0.030 | 1.604  (1.048-2.455) | 41  (10.99) | 14  (9.09) | 0.452 | 0.758  (0.369-1.559) | 0.49  (0.83) | 0.48  (0.86) | 0.964 | 0.992  (0.704-1.399) | 1.20  (2.02) | 2.01  (3.80) | 0.002 | 1.563  (1.176-2.077) |
| Recruitment |  |  |  |  |  |  |  |  |  |  |  |  |  |  |  |  |  |  |  |  |
| Outpatient | 2694  (26.95) | 1219  (30.08) | <.001 | 1.313  (1.192-1.445) | 4332  (43.34) | 2192  (54.08) | <.001 | 1.873  (1.714-2.047) | 515  (5.15) | 221  (5.45) | 0.255 | 1.114  (0.925-1.342) | 0.37  (0.71) | 0.45  (0.82) | <.001 | 1.334  (1.236-1.441) | 0.95  (1.82) | 1.57  (2.79) | <.001 | 1.749  (1.625-1.883) |
| Inpatient | 842  (31.87) | 319  (34.94) | 0.013 | 1.250  (1.047-1.492) | 1347  (50.98) | 551  (60.35) | <.001 | 1.739  (1.464-2.065) | 190  (7.19) | 64  (7.01) | 0.754 | 1.053  (0.762-1.455) | 0.46  (0.81) | 0.51  (0.84) | 0.006 | 1.202  (1.054-1.371) | 1.29  (2.18) | 1.82  (2.91) | <.001 | 1.562  (1.378-1.770) |
| Sedation use |  |  |  |  |  |  |  |  |  |  |  |  |  |  |  |  |  |  |  |  |
| No | 224  (32.32) | 96  (25.88) | 0.375 | 0.833  (0.556-1.248) | 332  (47.91) | 137  (36.93) | 0.036 | 0.669  (0.460-0.974) | 65  (9.38) | 22  (5.93) | 0.335 | 0.713  (0.359-1.419) | 0.44  (0.74) | 0.39  (0.77) | 0.624 | 1.082  (0.789-1.483) | 1.15  (1.99) | 1.03  (2.47) | 0.241 | 1.278  (0.848-1.925) |
| Yes | 3312  (27.73) | 1442  (31.38) | <.001 | 1.335  (1.224-1.456) | 5347  (44.76) | 2606  (56.71) | <.001 | 1.968  (1.814-2.134) | 640  (5.36) | 263  (5.72) | 0.089 | 1.155  (0.979-1.362) | 0.39  (0.73) | 0.47  (0.83) | <.001 | 1.320  (1.234-1.413) | 1.01  (1.90) | 1.66  (2.84) | <.001 | 1.748  (1.640-1.864) |
| Physician age, y |  |  |  |  |  |  |  |  |  |  |  |  |  |  |  |  |  |  |  |  |
| ≤40 | 1412  (27.19) | 419  (31.27) | <.001 | 1.377  (1.186-1.600) | 2291  (44.12) | 687  (51.27) | <.001 | 1.625  (1.414-1.868) | 272  (5.24) | 94  (7.01) | 0.038 | 1.340  (1.017-1.765) | 0.39  (0.74) | 0.47  (0.84) | <.001 | 1.350  (1.197-1.524) | 1.06  (2.06) | 1.46  (2.54) | <.001 | 1.578  (1.400-1.778) |
| ＞40 | 2124  (28.53) | 1119  (30.86) | <.001 | 1.309  (1.194-1.436) | 3388  (45.51) | 2056  (56.7) | <.001 | 2.031  (1.860-2.218) | 433  (5.82) | 191  (5.27) | 0.794 | 0.976  (0.815-1.170) | 0.39  (0.73) | 0.46  (0.82) | <.001 | 1.253  (1.160-1.353) | 0.99  (1.79) | 1.67  (2.91) | <.001 | 1.767  (1.644-1.900) |
| Years of endoscopic experience |  |  |  |  |  |  |  |  |  |  |  |  |  |  |  |  |  |  |  |  |
| ≤5 years | 261  (23.92) | 200  (29.07) | 0.420 | 1.126  (0.844-1.501) | 449  (41.15) | 297  (43.17) | 0.965 | 1.006  (0.774-1.307) | 44  (4.03) | 51  (7.41) | 0.166 | 1.472  (0.852-2.541) | 0.33  (0.70) | 0.43  (0.78) | 0.384 | 1.114  (0.874-1.420) | 0.96  (1.91) | 1.03  (1.91) | 0.907 | 0.987  (0.800-1.219) |
| 5-10 years | 595  (24.35) | 310  (23.99) | 0.901 | 0.986  (0.796-1.222) | 983  (40.22) | 513  (39.71) | 0.930 | 0.992  (0.822-1.197) | 147  (6.01) | 56  (4.33) | 0.158 | 0.752  (0.506-1.117) | 0.33  (0.66) | 0.35  (0.74) | 0.487 | 1.066  (0.891-1.275) | 0.92  (1.93) | 1.00  (2.31) | 0.024 | 1.225  (1.027-1.462) |
| ≥10 years | 2680  (29.44) | 1028  (34.43) | <.001 | 1.327  (1.189-1.481) | 4247  (46.65) | 1933  (64.74) | <.001 | 2.530  (2.270-2.819) | 514  (5.65) | 178  (5.96) | 0.102 | 1.192  (0.966-1.472) | 0.41  (0.75) | 0.52  (0.86) | <.001 | 1.285  (1.188-1.389) | 1.05  (1.90) | 2.01  (3.10) | <.001 | 1.833  (1.705-1.971) |
| Endoscopy volume |  |  |  |  |  |  |  |  |  |  |  |  |  |  |  |  |  |  |  |  |
| ≤5000 | 495  (24.89) | 443  (24.96) | 0.798 | 1.022  (0.865-1.208) | 803  (40.37) | 703  (39.61) | 0.683 | 0.969  (0.835-1.125) | 111  (5.58) | 99  (5.58) | 0.792 | 0.960  (0.707-1.303) | 0.33  (0.67) | 0.35  (0.72) | 0.374 | 1.065  (0.927-1.223) | 0.85  (1.64) | 0.92  (1.96) | 0.105 | 1.117  (0.977-1.278) |
| 5000-10000 | 702  (25.67) | 707  (36.00) | <.001 | 1.639  (1.388-1.936) | 1170  (42.78) | 1253  (63.8) | <.001 | 2.651  (2.262-3.107) | 140  (5.12) | 112  (5.70) | 0.145 | 1.279  (0.919-1.781) | 0.36  (0.71) | 0.54  (0.88) | <.001 | 1.487  (1.318-1.679) | 1.10  (2.24) | 2.13  (3.33) | <.001 | 2.011  (1.781-2.270) |
| ≥10000 | 2339  (29.56) | 388  (31.62) | 0.122 | 1.126  (0.969-1.309) | 3706  (46.83) | 787  (64.14) | <.001 | 2.408  (2.079-2.789) | 454  (5.74) | 74  (6.03) | 0.527 | 1.094  (0.829-1.444) | 0.41  (0.75) | 0.48  (0.86) | 0.017 | 1.150  (1.025-1.290) | 1.03  (1.84) | 1.78  (2.75) | <.001 | 1.714  (1.556-1.888) |
| AI Assistance |  |  |  |  |  |  |  |  |  |  |  |  |  |  |  |  |  |  |  |  |
| No | 963  (26.06) | 509  (30.05) | <.001 | 1.356  (1.160-1.586) | 1588  (42.97) | 921  (54.37) | <.001 | 2.116  (1.830-2.446) | 208  (5.63) | 100  (5.90) | 0.159 | 1.233  (0.921-1.650) | 0.36  (0.72) | 0.44  (0.81) | <.001 | 1.272  (1.122-1.442) | 0.99  (1.93) | 1.53  (2.83) | <.001 | 1.734  (1.548-1.942) |
| Yes | 2573  (28.77) | 1029  (31.45) | <.001 | 1.257  (1.136-1.391) | 4091  (45.75) | 1822  (55.68) | <.001 | 1.756  (1.597-1.931) | 497  (5.56) | 185  (5.65) | 0.649 | 1.046  (0.861-1.272) | 0.40  (0.74) | 0.47  (0.84) | <.001 | 1.291  (1.193-1.396) | 1.03  (1.90) | 1.65  (2.81) | <.001 | 1.704  (1.581-1.838) |

AOR: Adjusted odds ratio; CI: Confidence interval.
