# Supplementary material for: Exploring the influence of endoscopist characteristics and artificial intelligence on adenoma detection in colonoscopy
Source: Front Med (Lausanne). 2026 Jan 12;12:1720617. doi: 10.3389/fmed.2025.1720617 (PMC12833059; doi:10.3389/fmed.2025.1720617)
Supplement: Supplementary file 2 [file Table_2.docx]

**Table S2. Subgroup analysis of the effect of AI on endoscopists’ detection rates**

| Variables | ADR | | | | PDR | | | | AA | | | | APC | | | | PPC | | | |
| --- | --- | --- | --- | --- | --- | --- | --- | --- | --- | --- | --- | --- | --- | --- | --- | --- | --- | --- | --- | --- |
|  | Without AI | AI-assisted | *P* | AOR (95%CI) | Without AI | AI-assisted | *P* | AOR (95%CI) | Without AI | AI-assisted | *P* | AOR (95%CI) | Without AI | AI-assisted | *P* | AOR (95%CI) | Without AI | AI-assisted | *P* | AOR (95%CI) |
| Endoscopist gender |  |  |  |  |  |  |  |  |  |  |  |  |  |  |  |  |  |  |  |  |
| Male | 963  (26.06) | 2573  (28.77) | 0.001 | 1.166  (1.064-1.278) | 1588  (42.97) | 4091  (45.75) | 0.002 | 1.136  (1.046-1.234) | 208  (5.63) | 497  (5.56) | 0.841 | 0.983  (0.827-1.167) | 0.36  (0.72) | 0.40  (0.74) | 0.007 | 1.106  (1.028-1.190) | 0.99  (1.93) | 1.03  (1.90) | 0.496 | 1.025  (0.954-1.101) |
| Female | 509  (30.05) | 1029  (31.45) | 0.439 | 1.055  (0.921-1.209) | 921  (54.37) | 1822  (55.68) | 0.714 | 1.025  (0.899-1.168) | 100  (5.90) | 185  (5.65) | 0.956 | 0.993  (0.764-1.289) | 0.44  (0.81) | 0.47  (0.84) | 0.442 | 1.041  (0.939-1.155) | 1.53  (2.83) | 1.65  (2.81) | 0.588 | 1.028  (0.930-1.1387) |
| Physician age, y |  |  |  |  |  |  |  |  |  |  |  |  |  |  |  |  |  |  |  |  |
| ≤40 | 481  (26.46) | 1350  (28.63) | 0.062 | 1.134  (0.994-1.295) | 796  (43.78) | 2182  (46.28) | 0.015 | 1.160  (1.029-1.308) | 107  (5.89) | 259  (5.49) | 0.406 | 0.901  (0.704-1.152) | 0.38  (0.76) | 0.41  (0.76) | 0.132 | 1.086  (0.976-1.208) | 1.11  (2.16) | 1.16  (2.18) | 0.194 | 1.071  (0.966-1.188) |
| >40 | 991  (27.74) | 2252  (30.03) | 0.009 | 1.133  (1.032-1.243) | 1713  (47.96) | 3731  (49.75) | 0.077 | 1.080  (0.992-1.177) | 201  (5.63) | 423  (5.64) | 0.883 | 1.013  (0.849-1.210) | 0.39  (0.74) | 0.42  (0.77) | 0.015 | 1.094  (1.017-1.176) | 1.19  (2.31) | 1.22  (2.20) | 0.684 | 1.015  (0.944-1.092) |
| Years of endoscopic experience |  |  |  |  |  |  |  |  |  |  |  |  |  |  |  |  |  |  |  |  |
| ≤5 years | 176  (26.47) | 285  (25.58) | 0.800 | 0.968  (0.755-1.242) | 269  (40.45) | 477  (42.82) | 0.484 | 1.083  (0.866-1.356) | 39  (5.86) | 56  (5.03) | 0.706 | 0.913  (0.567-1.469) | 0.38  (0.76) | 0.36  (0.72) | 0.690 | 0.957  (0.773-1.186) | 1.01  (2.05) | 0.97  (1.82) | 0.366 | 0.911  (0.745-1.115) |
| 5-10 years | 231  (23.22) | 674  (24.59) | 0.216 | 1.124  (0.934-1.352) | 388  (38.99) | 1108  (40.42) | 0.143 | 1.129  (0.960-1.327) | 54  (5.43) | 149  (5.44) | 0.626 | 1.088  (0.775-1.528) | 0.32  (0.67) | 0.34  (0.70) | 0.393 | 1.069  (0.917-1.246) | 0.91  (1.92) | 0.97  (2.12） | 0.299 | 1.086  (0.930-1.268) |
| ≥10 years | 1065  (28.55) | 2643  (31.62) | <.001 | 1.164  (1.065-1.272) | 1852  (49.65) | 4328  (51.78) | 0.025 | 1.099  (1.012-1.194) | 215  (5.76) | 477  (5.71) | 0.688 | 0.966  (0.815-1.145) | 0.40  (0.76) | 0.45  (0.79) | 0.002 | 1.113  (1.039-1.192) | 1.25  (2.38) | 1.30  (2.26) | 0.429 | 1.028  (0.961-1.099) |
| Endoscopy volume |  |  |  |  |  |  |  |  |  |  |  |  |  |  |  |  |  |  |  |  |
| ≤5000 | 277  (25.65) | 661  (24.63) | 0.913 | 0.990  (0.831-1.180) | 430  (39.81) | 1076  (40.09) | 0.413 | 1.068  (0.912-1.250) | 62  (5.74) | 148  (5.51) | 0.972 | 1.006  (0.729-1.388) | 0.36  (0.73) | 0.33  (0.68) | 0.602 | 0.962  (0.831-1.114) | 0.92  (1.84) | 0.87  (1.78) | 0.977 | 1.002  (0.867-1.158) |
| 5000-10000 | 391  (27.30) | 1018  (31.16) | 0.029 | 1.178  (1.017-1.366) | 699  (48.81) | 1724  (52.77) | 0.058 | 1.141  (0.996-1.308) | 70  (4.89) | 182  (5.57) | 0.574 | 1.087  (0.812-1.455) | 0.40  (0.79) | 0.45  (0.79) | 0.270 | 1.067  (0.951-1.196) | 1.44  (2.92) | 1.57  (2.74) | 0.425 | 1.048  (0.934-1.177) |
| ≥10000 | 80  (27.94) | 1923  (30.70) | 0.010 | 1.145  (1.033-1.268) | 1380  (47.95) | 3113  (49.70) | 0.087 | 1.086  (0.988-1.193) | 176  (6.12) | 352  (5.62) | 0.280 | 0.900  (0.743-1.090) | 0.39  (0.73) | 0.44  (0.78) | 0.004 | 1.123  (1.037-1.216) | 1.11  (2.01) | 1.14  (2.00) | 0.696 | 1.015  (0.942-1.094) |

AOR: Adjusted odds ratio; CI: Confidence interval.
