# Supplementary figures and images for: Exploring the influence of endoscopist characteristics and artificial intelligence on adenoma detection in colonoscopy
Source: Front Med (Lausanne). 2026 Jan 12;12:1720617. doi: 10.3389/fmed.2025.1720617 (PMC12833059; doi:10.3389/fmed.2025.1720617)

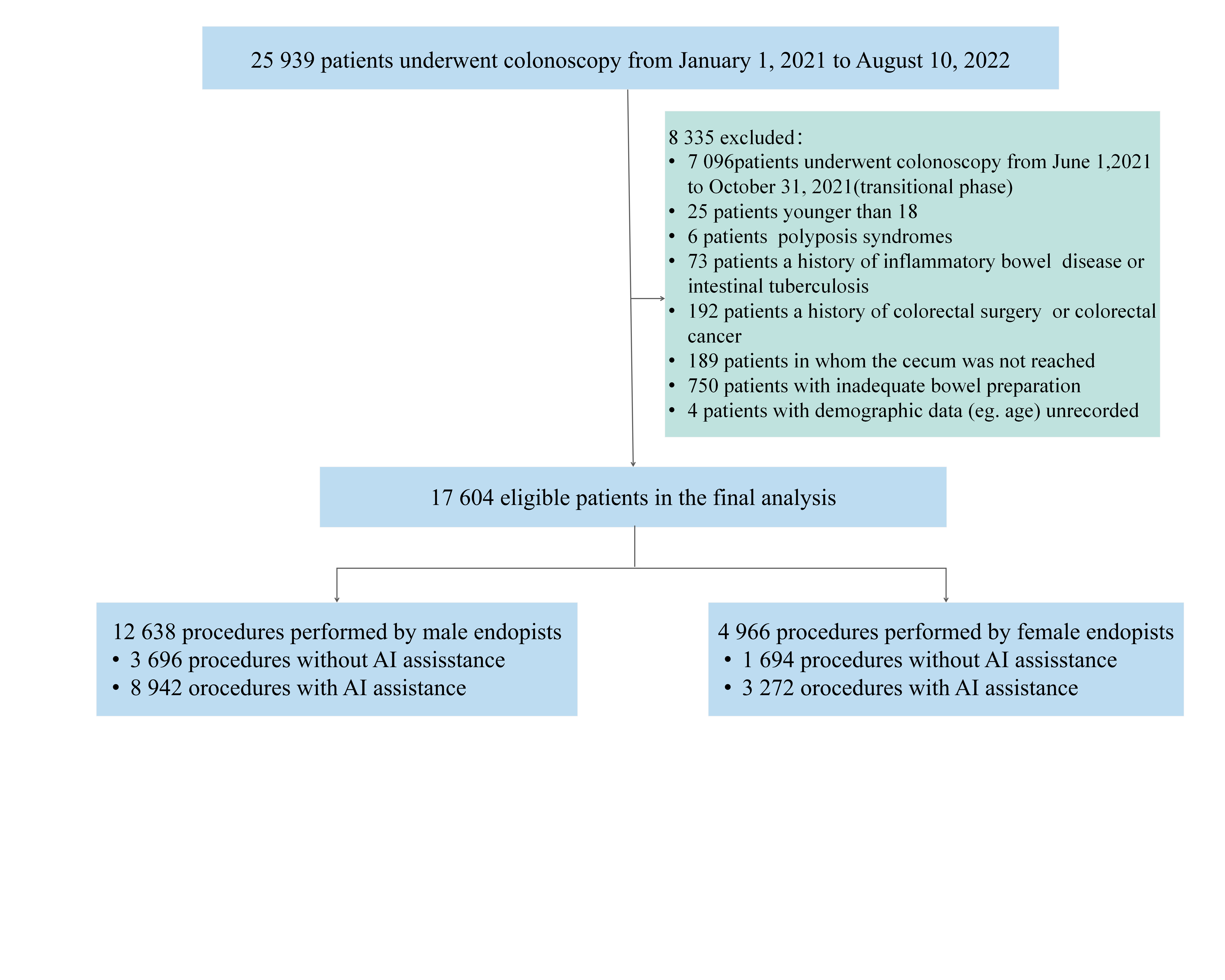

Supplement: Supplementary file 4 [file Image_1.tif]
